# Metropolis-Hastings algorithm in joint-attention naming game: experimental semiotics study

**DOI:** 10.3389/frai.2023.1235231

**Published:** 2023-12-05

**Authors:** Ryota Okumura, Tadahiro Taniguchi, Yoshinobu Hagiwara, Akira Taniguchi

**Affiliations:** ^1^Graduate School of Information Science and Engineering, Ritsumeikan University, Kusatsu, Japan; ^2^College of Information Science and Engineering, Ritsumeikan University, Kusatsu, Japan; ^3^Research Organization of Science and Technology, Ritsumeikan University, Kusatsu, Japan

**Keywords:** symbol emergence, experimental semiotics, naming game, probabilistic generative models, Bayesian inference

## Abstract

We explore the emergence of symbols during interactions between individuals through an experimental semiotic study. Previous studies have investigated how humans organize symbol systems through communication using artificially designed subjective experiments. In this study, we focused on a joint-attention-naming game (JA-NG) in which participants independently categorized objects and assigned names while assuming their joint attention. In the Metropolis-Hastings naming game (MHNG) theory, listeners accept provided names according to the acceptance probability computed using the Metropolis-Hastings (MH) algorithm. The MHNG theory suggests that symbols emerge as an approximate decentralized Bayesian inference of signs, which is represented as a shared prior variable if the conditions of the MHNG are satisfied. This study examines whether human participants exhibit behavior consistent with the MHNG theory when playing the JA-NG. By comparing human acceptance decisions of a partner's naming with acceptance probabilities computed in the MHNG, we tested whether human behavior is consistent with the MHNG theory. The main contributions of this study are twofold. First, we reject the null hypothesis that humans make acceptance judgments with a constant probability, regardless of the acceptance probability calculated by the MH algorithm. The results of this study show that the model with acceptance probability computed by the MH algorithm predicts human behavior significantly better than the model with a constant probability of acceptance. Second, the MH-based model predicted human acceptance/rejection behavior more accurately than four other models (i.e., Constant, Numerator, Subtraction, Binary). Among the models compared, the model using the MH algorithm, which is the only model with the mathematical support of decentralized Bayesian inference, predicted human behavior most accurately, suggesting that symbol emergence in the JA-NG can be explained by the MHNG.

## 1 Introduction

Humans can create and communicate through symbol systems that involve assigning meanings to signs. This semiotic process does not rely on predetermined definitions of the meanings of the symbols but rather emerges gradually through semiotic communication and perceptual experiences. This phenomenon is known as symbol emergence (Taniguchi et al., 2016, 2018). Understanding the cognitive capabilities and the social and cognitive dynamics that support symbol emergence is crucial in comprehending the dynamic property of language.

Numerous experimental semiotic studies have been conducted to investigate how humans organize symbol systems through communication (Galantucci, 2005; Healey et al., 2007; Scott-Phillips et al., 2009). These studies demonstrated that humans can build communication systems from scratch (Quinn, 2001; Galantucci, 2005; Healey et al., 2007; Scott-Phillips et al., 2009; Roberts, 2010). Additionally, computational-model-based studies in experimental semiotics, such as those by Kirby et al. (2008), Cornish (2010); and Navarro et al. (2018) validate the effectiveness of iterated learning models. Iterated learning is a process in which an individual acquires a behavior by observing a similar behavior in another individual who acquired it in the same way (Kirby et al., 2008). However, iterated learning is not an explanatory principle that answers the question of whether the emergence of a symbol system improves the environmental adaptation of a group of agents. Iterated learning does not have a theoretical connection to explanatory theories about human perceptual systems. By contrast, symbol emergence based on the Metropolis-Hastings naming game (MHNG), which is the focus of this study, is closely related to predictive coding and free-energy principle (Friston, 2010; Hohwy, 2013; Friston et al., 2021), which are often referred to as the general principle of cognition. In this context, Taniguchi et al. hypothesized that symbol emergence could be viewed as a collective predictive coding by a group of agents (Taniguchi, 2023; Taniguchi et al., 2023).

Many studies focused on computational models that represent symbol emergence systems. Pioneering studies have used naming games, in which remote robots share symbols to represent objects and variants of referential games (Cangelosi and Parisi, 1998; Steels, 1999, 2015; Kirby, 2002). More recently, deep-learning-based referential games have been extensively used to study emergent communication (Havrylov and Titov, 2017; Lazaridou et al., 2017; Evtimova et al., 2018; Bouchacourt and Baroni, 2019). Referential and naming games, often referred to as variants of the Lewis-style signaling game, have also been used to achieve compositionality in languages (Kottur et al., 2017; Choi et al., 2018; Ren et al., 2020; Mu and Goodman, 2021). Generally, in these games, a speaker sends a message to a listener who indicates the object intended by the speaker. After the communication, reward feedback is provided to the agents, and they update their parameters. The reward feedback precedes joint attention in this approach.

However, in the development of human infants, joint attention, which is acquired at around nine months of age, is well-known to precede tremendous progress in lexical acquisition and language development. Another notable concept is the naming game based on joint attention and the associated theoretical basis, called MHNG, in which each agent independently forms categories and shares signs associated with those categories through communication in the joint-attention naming game (JA-NG) (Hagiwara et al., 2019). This theory suggests that symbol emergence can be viewed as the approximate decentralized Bayesian inference of a posterior distribution over a shared latent variable conditioned on the observations of all agents participating in the communication. However, previous studies on experimental semiotics (Kirby et al., 2008; Cornish, 2010; Navarro et al., 2018) did not employ computational models that incorporate decentralized Bayesian inference over the entire system, including multiple agents.

In this study, our objective is to investigate whether the MHNG, which models symbol emergence as a decentralized Bayesian inference (Hagiwara et al., 2019; Taniguchi et al., 2023), can serve as a valid explanatory principle of symbol emergence between human individuals. MHNG involves computational agents playing the JA-NG, where agents independently form categories of objects and name them while assuming joint attention. Unlike the widely used Lewis signaling games (Lewis, 2008), the JA-NG does not involve any explicit reward feedback from the opponent after the naming process. In the MHNG, each agent decides whether to accept another agent's naming based on a probabilistic criterion calculated using the Metropolis-Hastings (MH) algorithm (Hastings, 1970). Consequently, symbol emergence occurs through a decentralized Bayesian inference.

Suppose people in the JA-NG follow a similar acceptance probability as observed in the MHNG. In this case, it can be inferred that they perform decentralized Bayesian inference as a whole system that includes multiple individuals involved in the emergence of symbols. The MHNG is a computational model in which agents play joint-attention naming games, and it uses the acceptance probability based on the MH algorithm to determine whether a listener agent accepts an incoming name proposed by another agent. Testing the hypothesis that humans use MH-based criteria to determine the acceptance of new names in the JA-NG is crucial to demonstrating the validity of the MHNG as an explanatory principle. If humans exhibit a behavior similar to that of the MHNG, their acceptance rate of incoming names should be correlated with the probability calculated using the MH algorithm. Thus, it can be concluded that humans make acceptance or rejection judgments in communication, following the MHNG principles to some extent. However, whether humans employ the same acceptance/rejection assessments in similar settings remains unclear.

This study aims to verify whether humans engage in decentralized Bayesian inference by conducting subject experiments similar to the JA-NG. We performed a communication experiment with human participants. The communication structure in the experiment resembled that of the JA-NG in a simulation experiment conducted by Hagiwara et al. (2019). We observed the acceptance or rejection assessments of participants and tested whether they used the acceptance probability calculated by the MHNG theory to a certain extent. Additionally, we evaluated whether the computational model using the MH algorithm predicted human behavior more accurately than four other comparative models: Constant, Numerator, Subtraction, and Binary.

The main contributions of this study are as follows:

- We verify whether human participants playing the JA-NG use the acceptance probability computed in the model based on the MH algorithm to a certain extent.
- We demonstrate that the model based on the MH algorithm outperforms the other four comparative computational models in predicting the acceptance behavior of participants in the JA-NG.

Statistical tests were conducted to examine our hypotheses. The results showed that the acceptance behavior of the human participants in the JA-NG can be modeled using the MH algorithm.

The remainder of this article is organized as follows. The next section provides an overview of the computational theory underlying this study. We then describe the setup of the communication experiment as well as the analysis and statistical test procedures in the Materials and Methods Section. The Results and Discussion Section presents our findings and corresponding interpretations. The final section concludes the article.

## 2 Preliminaries

In this section, we describe the JA-NG performed in the subject experiments and the interpersonal Gaussian mixture (Inter-GM), which is the assumed probabilistic model for analyzing the results of the subject experiments. Additionally, we describe the general interpersonal probabilistic generative model (Inter-PGM), whose concrete instance is Inter-GM, and the MHNG in which agents play the JA-NG using a specific acceptance probability based on the MH algorithms.

**Figure 1:**
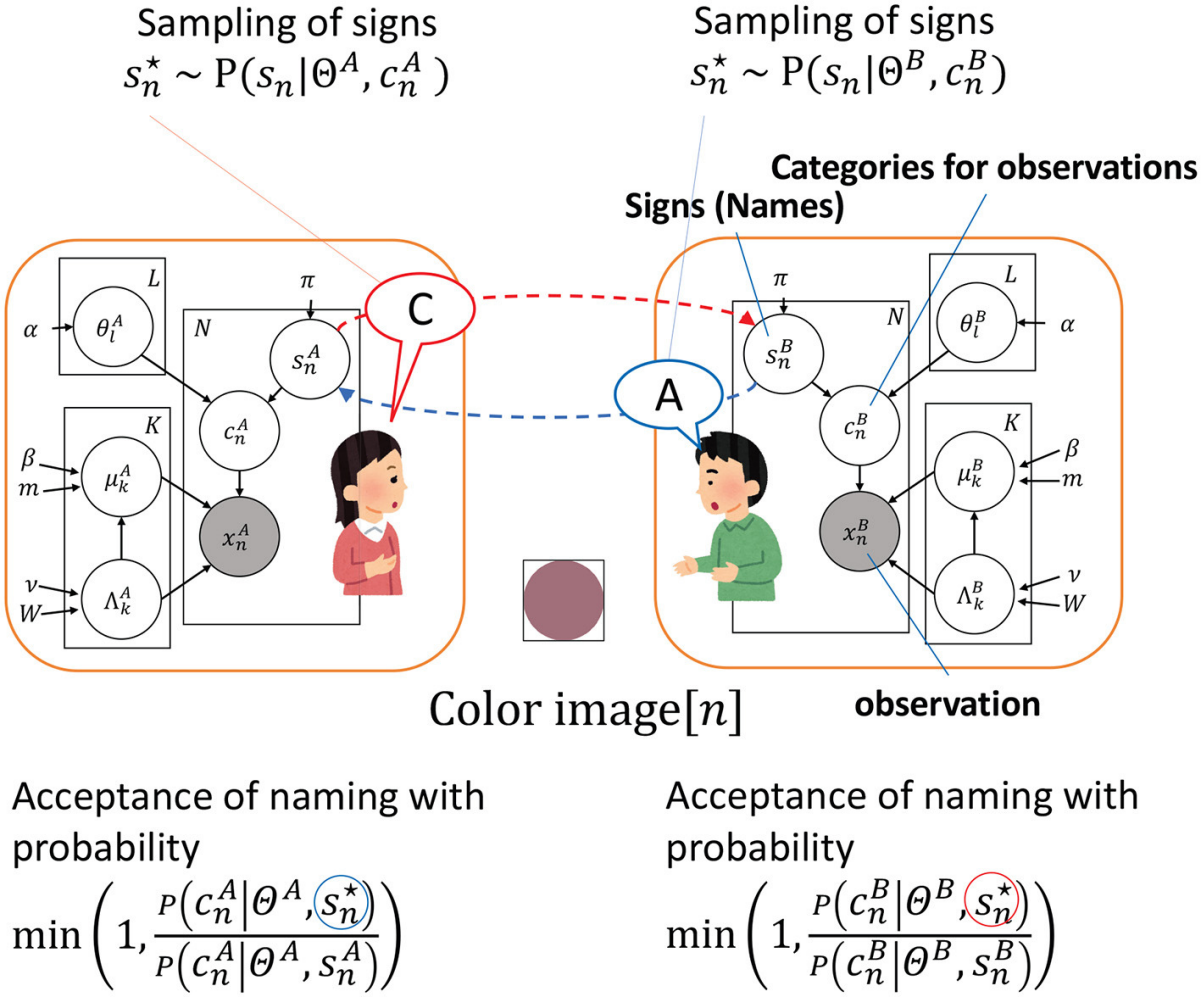
Illustration of the relationship between the communication game in the experiment and the probabilistic graphical model of the Inter-GM. The color images observed by the participants are labeled as $x_{n}^{A}$ and $x_{n}^{B}$, with the corresponding color classification results represented by $c_{n}^{A}$ and $c_{n}^{B}$. The images of the subjects are named by sampling a shared sign $s_{n}^{⋆}$, with signs sampled for the *n*-th object by A and B, which are labeled as $s_{n}^{A}$ and $s_{n}^{B}$, respectively. The red balloon is the sampled sign of A and the blue one is the sampled sign of B. The transmission of the sign through naming is depicted by the dashed red and blue lines. The variable Θ^*^ consists of *L* elements, specifically denoted as $\Theta^{*}={\left\{\theta_{l}^{*}\right\}}_{l=1,\ldots ,L}$. Similarly, the variable Φ^*^ encompasses *K* paired elements, with each pair consisting of $\mu_{k}^{*}$ and $\Lambda_{k}^{*}$, represented as $\Phi^{*}={\left\{\left(\mu_{k}^{*},\Lambda_{k}^{*}\right)\right\}}_{k=1,\ldots ,K}$.

Figure 1 illustrates the correspondence between the computational model (i.e., Inter-GM) and the communication experiment.

### 2.1 Joint-attention naming game

Two agents *A* and *B* play the JA-NG as detailed here. Specific variables are introduced in the following subsection.

1. Perception: Both the speaker and listener observe an object and update their perceptual state, such as a categorization result corresponding to the object based on their respective observations, assuming joint attention where two agents are looking at the same object.
2. Communication: The speaker gives the name to the object based on its perceptual state (e.g., the categorization result, and its own knowledge). The listener decides whether to accept the name.
3. Learning: After communication, the categorization results and knowledge are updated based on the results of the communication.
4. Turn-taking: The speaker and listener alternate their roles and repeat the above steps for all objects.

The JA-NG is a procedural description of the interaction between two agents and their learning process through the sharing of semiotic knowledge between them based on joint attention.

### 2.2 Inter-PGM and MH naming game

**Figure 2:**
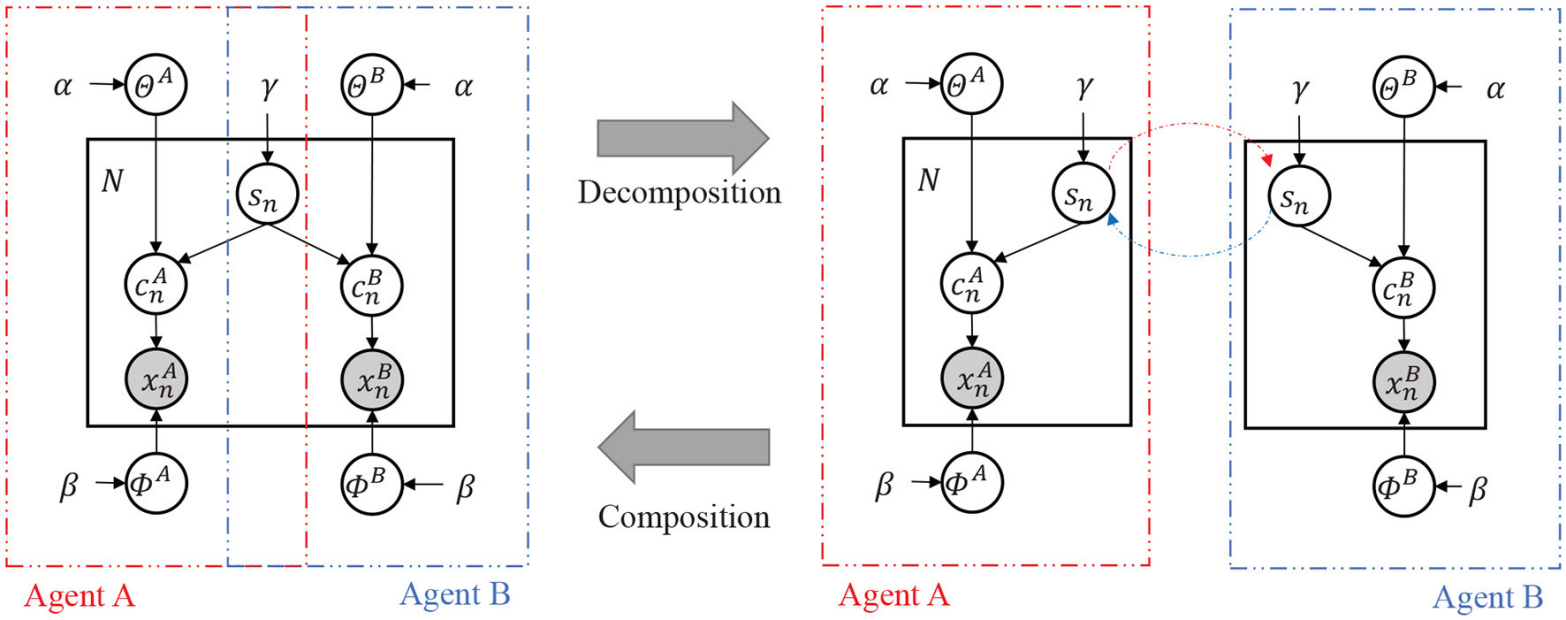
**(Left)** Probabilistic graphical model of the Inter-PGM. **(Right)** Decomposed illustration of the Inter-PGM.

We first define the variables related to the JA-NG and assume a conditional dependency between the variables by defining the Inter-PGM (Figure 2). Table 1 is an explanation of the variables in the Inter-PGM. An Inter-PGM is a general form of PGM that models symbol emergence using the JA-NG.

**Table 1:** Variables of the Inter-PGM and their explanations.

**Variable**	**Explanation**
*s*_*n*_	A sign, e.g., a name, for the *n*-th object
$c_{n}^{*}$	Perceptual state corresponding to the *n*-th object
$x_{n}^{*}$	Observation for the *n*-th object
Θ^*^	Parameter about the relations between signs and perceptual states
Φ^*^	Parameter about the relations between perceptual states and observations
α	A hyperparameter for Θ^*^
β	A hyperparameter for Φ^*^

Superscript *∈{*A, B*} refers to a specific agent.

The probability variables related to the JA-NG can be described using a probabilistic graphical model, as shown in Figure 2.

The generative process of the Inter-PGM is as follows:


(1) $$\begin{array}{ll} s_{n} & \sim P\left(s_{n}|\gamma \right)\;n=1,\ldots ,N \end{array}$$

(2) $$\begin{array}{ll} \Theta^{*} & \sim P\left(\Theta^{*}|\alpha \right) \end{array}$$

(3) $$\begin{array}{ll} \Phi^{*} & \sim P\left(\Phi^{*}|\beta \right) \end{array}$$

(4) $$\begin{array}{ll} c_{n}^{*} & \sim P\left(c_{n}^{*}|s_{n},\Theta^{*}\right)\;n=1,\ldots ,N \end{array}$$

(5) $$\begin{array}{ll} x_{n}^{*} & \sim P\left(x_{n}^{*}|c_{n}^{*},\Phi^{*}\right)\;n=1,\ldots ,N \end{array}$$

where $x_{n}^{*}$ represents the observed information, $c_{n}^{*}$ represents the category to which $x_{n}^{*}$ is classified, that is, perceptual state, and $s_{n}^{*}$ represents the sign of $x_{n}^{*}$, and *∈{*A, B*}.

A PGM can be decomposed into two parts corresponding to the two agents using the Neuro-SERKET framework (Taniguchi et al., 2020) in the inference process. found that a certain type of language game can be regarded as a decentralized inference process for an Inter-PGM, and Taniguchi et al. (2023) formulated this idea as the MHNG.

The MH naming game is a special case of the JA-NG (Taniguchi et al., 2023). The JA-NG becomes the MHNG upon satisfying the following conditions:

1. The speaker (*Sp*) selects the name $s_{n}^{⋆}$ by sampling from the posterior distribution $P\left(s_{n}|\Theta^{Sp},c_{n}^{Sp}\right)$.
2. The listener (*Li*) determines acceptance of sign $s_{n}^{⋆}$ using the probability $r^{MH}=\min\left(1,\frac{P(c_{n}^{Li}|\Theta^{Li},s_{n}^{⋆})}{P(c_{n}^{Li}|\Theta^{Li},s_{n}^{Li})}\right)$.
3. The agents update its internal variables $c_{n}^{*},\Theta^{*},\Phi^{*}$ using Bayesian inference appropriately.

It is theoretically guaranteed that the MHNG is an approximate decentralized Bayesian inference of shared representations, that is, $P\left({\left\{s_{n}\right\}}_{n=1,\ldots ,N}|{\left\{x_{n}^{A},x_{n}^{B}\right\}}_{n=1,\ldots ,N}\right)$ and the internal representations and the knowledge of each agent. Specifically, internal representations are characterized by the local parameters $c_{n}^{*}$, while knowledge is defined by the global parameters Θ^*^ and Φ^*^ in Figure 2. These global parameters represent the relationship between observations and internal representations, and the relationship between names and internal representations, respectively. For more details, please refer to the original article (Taniguchi et al., 2023).

### 2.3 Interpersonal Gaussian mixture

We used Inter-GM, which was tailored to fit the observations, that is, the color information used in our experiment. Hagiwara et al. (2019, 2022) proposed inter-DM and inter-MDM models in which agents observed bag-of-features representations, that is, histograms. They formed individual categories using a Dirichlet mixture and shared signs linked to the formed categories through communication. Inter-GM is a modified version of inter-DM in which the part that formed categories using a Dirichlet mixture is replaced by a Gaussian mixture for categorizing multidimensional continuous real-valued vectors.

The Inter-GM generative process is as follows:


(6) $$\begin{array}{ll} s_{n} & \sim \text{Cat}\left(s_{n}|\pi \right)\;n=1,\ldots ,N \end{array}$$

(7) $$\begin{array}{ll} \mu_{k}^{*},\Lambda_{k}^{*} & \sim N\left(\mu_{k}^{*}|m,{\left(\beta \Lambda_{k}^{*}\right)}^{-1}\right)W\left(\Lambda_{k}^{*}|\nu ,W\right)\;k=1,\ldots ,K \end{array}$$

(8) $$\begin{array}{ll} \theta_{l}^{*} & \sim \text{Dir}\left(\theta_{l}^{*}|\alpha \right)\;l=1,\ldots ,L \end{array}$$

(9) $$\begin{array}{ll} c_{n}^{*} & \sim \text{Cat}\left(c_{n}^{*}|\theta_{s_{n}}^{*}\right)\;n=1,\ldots ,N \end{array}$$

(10) $$\begin{array}{ll} x_{n}^{*} & \sim N\left(x_{n}^{*}|\mu_{c_{n}}^{*},{\left(\Lambda_{c_{n}}^{*}\right)}^{-1}\right)\;n=1,\ldots ,N \end{array}$$

Cat(*) is the categorical distribution, N(*) is the Gaussian distribution, W(*) is the Wishart distribution, and Dir(*) is the Dirichlet distribution. The parameters for the Gaussian mixture model (GMM) ${\left\{\mu_{k}^{*},\Lambda_{k}^{*}\right\}}_{k=1,\ldots ,K}$ correspond to Φ^*^ and ${\left\{\theta_{l}^{*}\right\}}_{l=1,\ldots ,L}$ corresponds to Θ^*^ in Inter-PGM (Figure 2) respectively.

In the MHNG, after observing (or sampling) $s_{n}^{*}$, the probabilistic variables for each agent become independent, and the parameters for each agent can be inferred using ordinal approximate Bayesian inference schemes. We applied Gibbs sampling, a widely used Markov chain Monte Carlo approximate Bayesian inference procedure (Bishop and Nasrabadi, 2006), to sample the parameters $\mu_{k}^{*}$, $\Lambda_{k}^{*}$, $c_{n}^{*}$, and Θ^*^.

In the MHNG, the sign *s*_*n*_ is inferred by agents A and B through an alternative sampling of the sign *s*_*n*_ from each other, and acceptance based on the acceptance probability of the MH algorithm $r_{n}^{MH}=\min\left(1,\frac{P(c_{n}^{Li}|\Theta^{Li},s_{n}^{⋆})}{P(c_{n}^{Li}|\Theta^{Li},s_{n}^{Li})}\right)$ for the sign of the other agent where $\Theta^{Li}={\left\{\theta_{l}^{Li}\right\}}_{l=1,\ldots ,L}$ is inferred using $c_{n}^{Li}$ and $s_{n}^{⋆}$.

**Figure 3:**
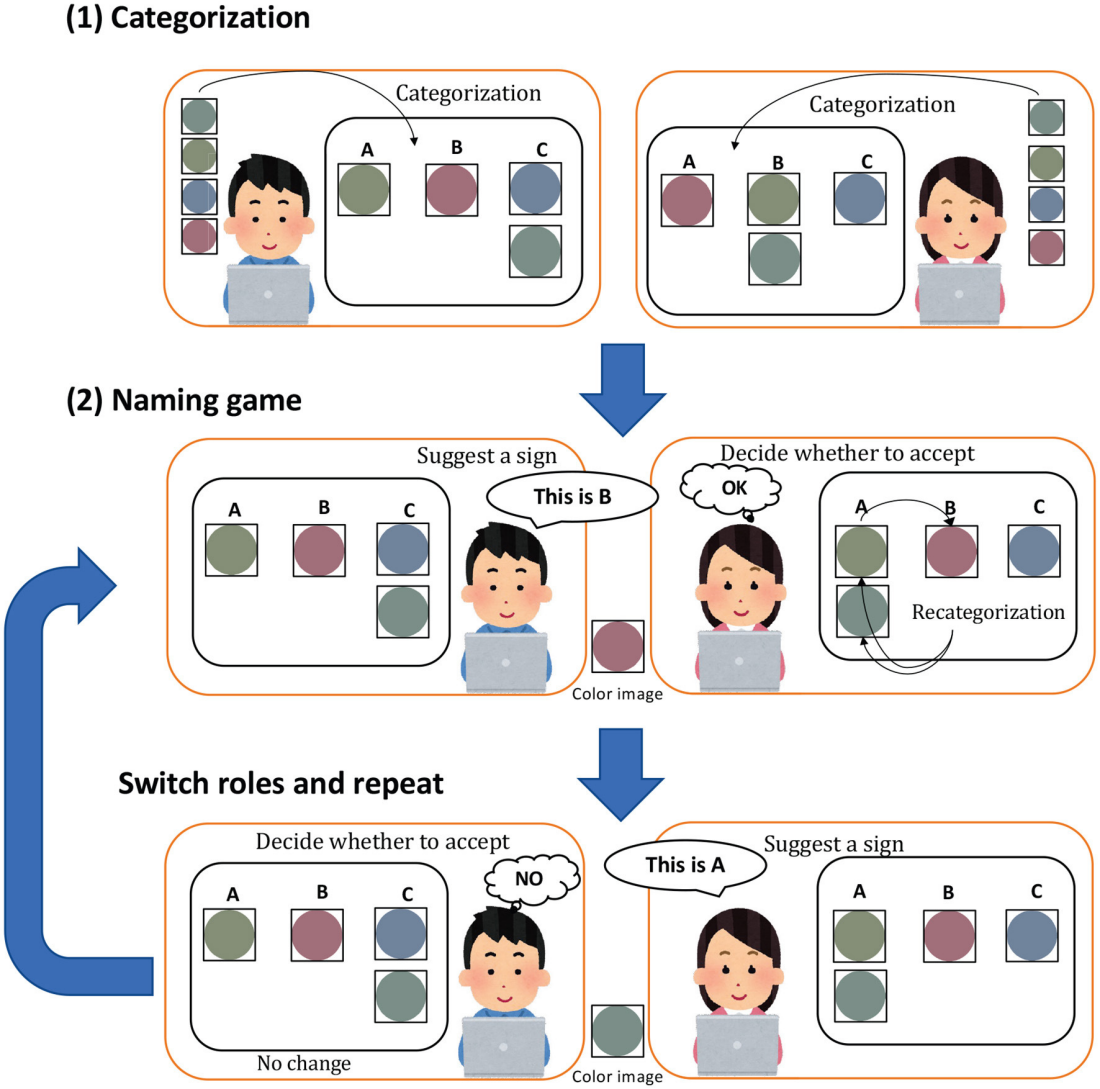
Flow of the subject experiment. In (1) categorization, participants categorize the given image. In (2) naming game, the speaker names the image by selecting any name from A to E, and the listener, decides whether to accept or reject the proposed name by pressing a button. Participants repeat the process, switching between the roles of speaker and listener.

The acceptance probability estimated from the categorization results (see Figure 3) and the actual acceptance/rejection decisions were recorded to investigate whether humans accept the proposals of their opponents based on the MH acceptance probability. The parameters Θ^*^ and Φ^*^ are inferred through Gibbs sampling using the categorization ${\left\{c_{n}^{*}\right\}}_{n=1,\ldots ,N}$ provided by the participants, along with their names $s_{n}^{*}$ and original observations $x_{n}^{*}$. The MH acceptance probability $r_{n}^{MH}$ is then calculated, where $s_{n}^{⋆}$ denotes the proposal of the opponent.

## 3 Materials and methods

### 3.1 Communication experiment

To investigate whether the acceptance of the speaker's proposals by a listener aligns with the acceptance probability calculated by the MH algorithm $r_{n}^{MH}$, we performed a communication experiment with human participants. Instead of the computational experiment described in Hagiwara et al. (2019), we performed a communication experiment with human participants that followed a methodology similar to that of experimental semiotics.

The experiment was conducted in pairs, referred to as participants A and B. Each pair followed the procedure outlined in Figure 3 and used separate personal computers (PCs). Participants were in different rooms and were not permitted to communicate directly using any alternative communication media.

**Figure 4:**
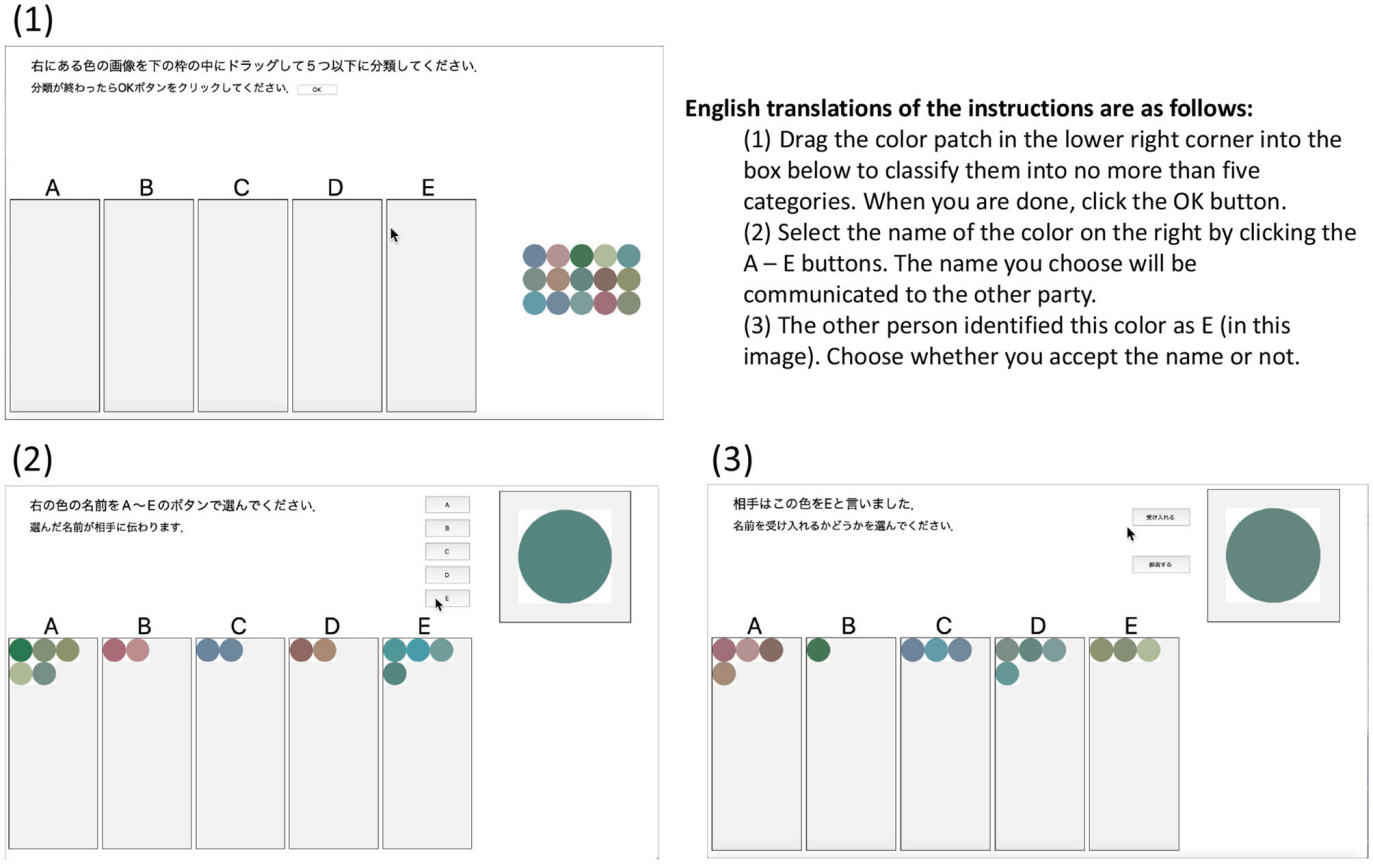
Screenshots of the experimental application in operation. (1) a view of the initial categorization phase, (2) the view of the speaker in the naming phase, and (3) the view of the listener when the listener receives a name for a color patch.

Figure 4 shows the user interface of the experimental application. (1) in Figure 4 shows the category classification screen that the participants first encountered, (2) shows the screen for the name, and (3) shows the screen for the listener. The procedure is detailed below.

Before starting the communication, each participant was instructed to classify 15 images into categories labeled A–E (**initialization**).

1. **Perception**: An image used in the initialization step is displayed to a speaker. In the experiment, the participants were asked to exhibit their perceptual state as a categorization result [(1) Categorization in Figure 3].
2. **Communication**: The speaker names the image by selecting any name from A to E. Participant B, the listener, decides whether to accept or reject the proposed name by pressing a button.
3. **Learning** (update categories and sign allocation): Participant B, as the listener, can modify their classification result after the acceptance/rejection decision.
4. **Turn-taking**: Steps from 1 to 3 correspond to (2) naming game as shown in Figure 3, and this game is repeated with participants switching their roles.

During the experiment, the participants repeated steps 2–4 15 times for each data sample, and the process was done three times. Therefore, each participant made 45 acceptance or rejection decisions per dataset.

**Figure 5:**
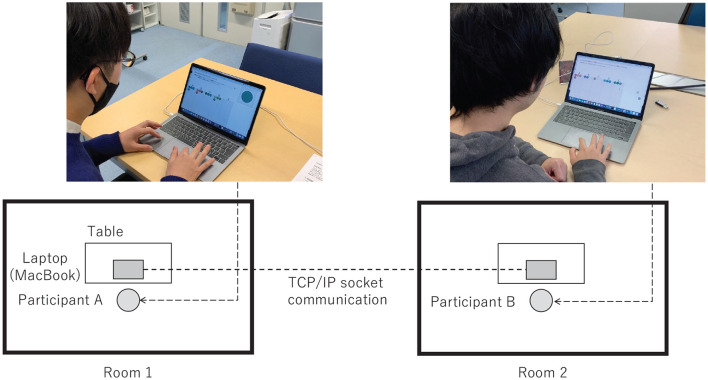
State of the actual experiment. The two participants used different PCs and were housed in separate rooms, and they communicated using socket communication.

The communication process involved proposing and accepting/rejecting names in steps 1 and 2. Each communication was completed when step 2 ended and the results were recorded each time. Participants could modify their classification results whenever desired; however, a prompt appeared if they attempted to alter the result after accepting/rejecting the proposal of their partner when playing the role of the listener. The participant pairs were housed in separate rooms, and the classification and communication were performed on PCs using a Python application, which communicated with the other PCs. The PCs used were 13-inch MacBooks. The brightness of the PCs was automatically adjusted to account for the possibility of different ambient lighting in each room. The images were presented in random order because the same images were used even after switching roles in step 3. Figure 5 shows a photograph of an actual experiment. Participants were given the following instructions:

- Work collaboratively with your counterpart to improve classification accuracy through interactions.
- Sharing names is crucial for effective communication.
- When you receive naming suggestions from your counterpart, decide whether to accept their naming or stick with your own to improve classification accuracy.

### 3.2 Computational model for analysis

We used the Inter-GM described in the Preliminaries section to analyze the behavioral data and predict the acceptance rate of the participants.

The hyperparameters used for the Inter-GM were *α* = (0.1, 0.1, 0.1, 0.1, 0.1)^T^, *β* = 1.0, *m* = (50, 0, 0)^T^, $W^{-1}=\left(\begin{array}{ccc} 200 & 0 & 0\\ 0 & 200 & 0\\ 0 & 0 & 200 \end{array}\right)$, *π* = (1/5, 1/5, 1/5, 1/5, 1/5)^T^, in an empirical manner.

### 3.3 Materials

For the experiment, 20 participants were recruited, forming 10 pairs. The female-to-male ratio was 6:14, and the minimum and maximum ages were 21 and 59, respectively. As the experiment used colors, the participants were verbally asked whether they were colorblind to ensure that colorblind participants were not included in the experiment. The study was initiated on 27 July 2022, and the recruitment of participants commenced on 15 December 2022. The experiment took place from 20 December 2022 to 25 January 2023. Throughout this period, the authors created and maintained the experimental data of the participants and correspondence tables. It is important to note that the correspondence tables were not accessed or used during the data analysis stage.

This study was approved by the Research Ethics Committee of Ritsumeikan University under approval number BKC-LSMH-2022-012. All the participants provided informed consent before participation.

To generate color images as stimuli, the CIE-L^*^U^*^V^*^ color space, which accurately represents the psychological distance perceived by humans, was used (Steels et al., 2005). In the CIE-L^*^U^*^V^*^ color space, L^*^ represents brightness and U^*^V^*^ represents hue. The details of the color images were as follows: (1) Pillow (PIL), a Python image processing library, was used to create images of colored circles^1^. (2) L^*^, U^*^, and V^*^ were sampled from three three-dimensional Gaussian distributions. (3) Two datasets, **hard** and **easy**, were prepared to observe the differences in communication according to difficulty levels: Dataset 1 was difficult to classify, and Dataset 2 was easy to classify. (4) The same images were shown to both participants and each dataset contained 15 images. (5) The Gaussian distribution to sample from was determined using a uniform distribution.

**Figure 6:**
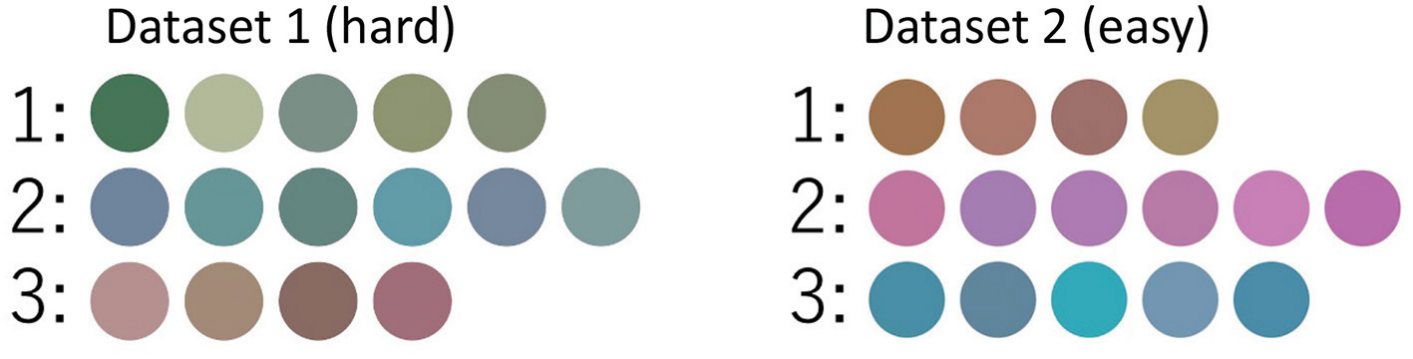
Images used in the communication experiment. The left image is Dataset 1 (hard), and the right image is Dataset 2 (easy). The images in the same row are generated from the same Gaussian distribution, and the numbers represent the Gaussian distribution number.

Table 2 lists the parameters for each Gaussian distribution. Each data point in the three-dimensional CIE-L^*^U^*^V^*^ color space was generated from a three-dimensional Gaussian distribution. Figure 6 shows images of Dataset 1 (**hard**), and Dataset 2 (**easy**).

**Table 2:** Parameters of the three Gaussian distributions generating the color patches used in the experiment.

	**Dataset 1 (hard)**	**Dataset 2 (easy)**
μ_1_	$\left(\begin{array}{c} 60\\ -10\\ 20 \end{array}\right)$	$\left(\begin{array}{c} 60\\ 30\\ 30 \end{array}\right)$
μ_2_	$\left(\begin{array}{c} 60\\ -20\\ -10 \end{array}\right)$	$\left(\begin{array}{c} 60\\ 30\\ -30 \end{array}\right)$
μ_3_	$\left(\begin{array}{c} 60\\ 20\\ 10 \end{array}\right)$	$\left(\begin{array}{c} 60\\ -30\\ -30 \end{array}\right)$
Σ	$\left(\begin{array}{ccc} 5^{2} & 0 & 0\\ 0 & 9^{2} & 0\\ 0 & 0 & 9^{2} \end{array}\right)$	$\left(\begin{array}{ccc} 5^{2} & 0 & 0\\ 0 & 10^{2} & 0\\ 0 & 0 & 10^{2} \end{array}\right)$

μ_*k*_ is the mean vector of the *k*-th three-dimensional Gaussian distribution. Σ = Λ^−1^ is the covariance matrix that is shared among the three Gaussian distributions.

### 3.4 Hypothesis testing 1

We investigated whether the decisions of people are affected by their acceptance probability using the acceptance probability based on the MH algorithm, although the decision does not completely comply with the theory. To investigate whether humans use the MH-based acceptance probability to a certain extent, that is, whether the actual acceptance probability correlates with the MH-based acceptance probability, we defined a biased Bernoulli distribution, $\text{Bern}\left(z_{n}|r_{n}=ar_{n}^{MH}+b\right)$. The Bernoulli distribution, Bern(*z*∣*r*), samples one with probability *r* and zero with probability 1−*r*. The weight parameter *a*, indicating the extent to which the inferred acceptance probability is used, and bias parameter *b*, indicating the degree to which acceptance occurs unconditionally, were used and estimated. If *a* = 1 and *b* = 0, the distribution becomes the original MH-based acceptance probability distribution, $\text{Bern}\left(z_{n}|r_{n}=r_{n}^{MH}\right)$. Specifically, variable *z*_*n*_ represents whether the participant accepted the given name, taking the value of 1 if accepted and 0 if rejected. The acceptance probability of a participant is denoted by *r*_*n*_.

The linear transformation was introduced to account for external social and cognitive factors that might affect acceptance probability. While the MHNG theory focuses on perceptual states and their relationship to signs, as shown in Figure 1, it does not consider other social or cognitive influences on acceptance probability. Factors such as respect for a counterpart or an authority gradient (Gluyas, 2015) could make it easier to accept another's proposal. Notably, to reduce the influence of these factors during the experiment, we conducted sessions with participants in separate rooms, ensuring that they could not see each other.

We tested the estimated parameter *a*, which model the relationship between the actual acceptance probability and MH-based acceptance probability $r_{n}^{MH}$. For acceptance and rejection, we assumed 1 and 0, respectively. Instead of calculating the correlation between the acceptance/rejection decision and *r*_*n*_, we used a conditional Bernoulli distribution.

Parameters *a* and *b* were determined using the maximum likelihood estimation. The maximum log-likelihood estimation of parameters *a* and *b* was performed using gradient descent. The original likelihood function is defined as


$$\begin{array}{l} L\left(a,b\right)=\overset{N}{\underset{n=1}{\prod }}\text{Bern}\left(z_{n}|r_{n}=ar_{n}^{MH}+b\right) \end{array}$$

To avoid the Bern parameter from going outside the domain, *a* and *b* were bounded to 0 ≤ *b* and *a*+*b* ≤ 1, respectively.

A hypothesis test was performed to test the statistical significance of the association between the $r_{n}^{MH}$ score and acceptance decisions made by human participants.

The null hypothesis *H*_0_ and alternative hypothesis *H*_1_ are as follows:

- *H*_0_: There is no association between the acceptance decision and $r_{n}^{MH}$, the MH-based acceptance probability. In other words, the human acceptance probability remains constant with respect to $r_{n}^{MH}$.
- *H*_1_: The acceptance probability is not constant, indicating that the acceptance probabilities calculated by the MH algorithm are more predictive of human judgment than random acceptance probabilities.

The test statistic is the coefficient of a (bounded) linear function that parameterizes the Bernoulli distribution and the acceptance probability as output. The test statistic was set as the coefficient of the regression fitted to the observed data â.

To estimate the sampling distribution of the test statistic, we used a randomized approach in which we randomly generated Bernoulli random variables with a fixed parameter and then fitted a linear model to obtain the coefficient *a* (i.e., the test statistic) from the null hypothesis^2^. The acceptance and rejection decisions were randomly sampled from the distribution by assuming *H*_0_, that is, $z_{n}\sim \text{Bern}\left(z|\bar{b}\right)$. The null distribution of the test statistics was estimated and the *p*-values were empirically calculated. By performing this 1,000 times, we obtained an estimate of the sampling distribution as a histogram, which we used to compute the *p*-value as the tail probability. $\bar{b}$ was determined from the behavior of all subjects using maximum likelihood estimation.

By assuming that the acceptance events occur with probability *r*_*n*_, we can compute the likelihood by fitting them to the Bernoulli distribution and multiplying them by the total number of given names *N*; that is, $L=\prod_{n=1}^{N}\text{Bern}\left(z_{n}|r_{n}=ar_{n}^{MH}+b\right)$

We performed sampling using $\text{Bern}\left(\bar{z}|\bar{b}\right)$ to obtain lists of test statistics *a* and created their cumulative distribution functions to conduct a statistical test. The following steps describe the process of obtaining the list of test statistics *a*: From the experimental results, we calculated the acceptance rate $\bar{b}=\frac{1}{N}\overset{N}{\underset{n=1}{\sum }}z_{n}$ for all participants or target participants across all trials. We sampled the acceptance or rejection of each round from the Bernoulli distribution $\text{Bern}\left(\bar{z}|\bar{b}\right)$ with the parameter $\bar{b}$ determined in the previous step, that is, ${\bar{z}}_{n}\sim \text{Bern}\left(\bar{z}|\bar{b}\right)\;\left(n\in 1,\ldots ,N\right)$. Parameter *a* was estimated using the maximum likelihood estimator for each sampling result and was added to the list of statistical quantities. This procedure was performed 1, 000 times and the sample distributions of *a* were obtained.

We computed the cumulative distribution function $P_{a}^{\prime }\left(a\right)=\frac{1}{L}\overset{L}{\underset{l=1}{\sum }}f\left(a_{l},a\right)$ from a list of obtained statistical values *a* represented as *a*_1_, *a*_2_, …, *a*_*L*_, where *L* = 1, 000. Similarly, we computed the cumulative distribution function $P_{b}^{\prime }\left(b\right)=\frac{1}{L}\overset{L}{\underset{l=1}{\sum }}f\left(b_{l},b\right)$ from a list of statistical values *b*, represented by *b*_1_, *b*_2_, …, *b*_*L*_. Here,


$$f(x,y)=\{\begin{array}{ll} 1, & x\geq y\\ 0, & x<y \end{array}$$

where *f*(*x, y*) represents a function that returns 1 if *x* exceeds or is equal to *y*, and 0 if *x* is below *y*. Because *r*_*MH*_ can be used if it is significantly greater than 0, *a* one-sided test was performed.

### 3.5 Hypothesis testing 2

In Test 2, we tested whether the model that used the MH algorithm, that is, the acceptance decision using $\text{Bern}\left(z_{n}|r_{n}^{MH}\right)$, was closer to the behavior of participants than that using several heuristic comparative models. We performed a test using the assessment of acceptance or rejection obtained from the results of the communication experiment, and the inferred acceptance probability was denoted as $r_{n}^{MH}$. We created a dataset consisting of distances between the behaviors of participants and the samples generated from the probabilities calculated by the five comparison models. These models were used to evaluate the acceptance and rejection. Subsequently, *U*-tests were conducted for each model.

Table 3 lists the comparative models used in this study. **Constant** accepts with a probability $\bar{b}$ calculated from the actual acceptance rate of the subject from the experimental results, which corresponds to the null hypothesis of Hypothesis testing 1. **MH** accepts with the inferred MH-based acceptance probability $r_{n}^{MH}$ from the experimental results. **Numerator** accepts with the acceptance probability being the numerator part of the $r_{n}^{MH}$ score, which represents the likelihood of the sign of the opponent using its own parameter. **Subtraction** calculates the difference between the numerator part of the $r_{n}^{MH}$ score representing the likelihood of the sign of the opponent using the parameter of the listener and the denominator part representing the likelihood of its own sign instead of the ratio in $r_{n}^{MH}$ score. Subsequently, it was transformed into a range of 0.0–1.0. **Binary** accepts with a probability of 0.1 if the inferred acceptance probability $r_{n}^{MH}$ is less than or equal to 0.5 and 0.9 if it exceeds 0.5.

**Table 3:** Details of each model.

**#**	**Model name**	**Acceptance probability formula**
1	Constant	$r_{n}^{1}=\bar{b}$
2	MH	$r_{n}^{2}=r_{n}^{MH}$
3	Numerator	$r_{n}^{3}=P\left(c_{n}^{Li}|\Theta^{Li},s_{n}^{⋆}\right)$
4	Subtraction	$r_{n}^{4}=\left(P\left(c_{n}^{Li}|\Theta^{Li},s_{n}^{⋆}\right)-P\left(c_{n}^{Li}|\Theta^{Li},s_{n}^{Li}\right)\right)/2+1/2$
5	Binary	$r_{n}^{5}=\{\begin{array}{ll} 0.1 & \left(r_{n}^{MH}\leq 0.5\right)\\ 0.9 & \left(r_{n}^{MH}>0.5\right) \end{array}$

To test the statistical significance of models *m* and *m*′ that make decisions regarding acceptance and rejection, hypothesis tests were performed as null and alternative hypotheses, respectively, as follows:

- *H*_0_: ${\text{Prec}}_{m}={\text{Prec}}_{m^{\prime }}$. The models *m* and *m*′ predict the behavior of participants at the same level.
- *H*_1_: ${\text{Prec}}_{m}>{\text{Prec}}_{m^{\prime }}$. The model *m* predicts the behavior of participants more accurately than the model *m*′.

Here, Prec_*m*_ is the rate at which the model *m* could predict the acceptance or rejection decisions of participants, that is, precision.

We sampled 100 data points for the pseudo-experimental results of each comparison model using computer simulations. The pseudo-experimental results for each comparison model were sampled from the Bernoulli distribution with the parameter of acceptance probability $r_{\left(j,i\right)}^{m}$ for subject *j* of model *m* in the *i*th communication trial and labeled 1 for acceptance and 0 for rejection. The *p* values were calculated using a *U*-test, and the significance level was set at 0.001.


$$\begin{array}{l} z_{\left(j,i\right)}^{m}\sim \text{Bern}\left(z|r_{\left(j,i\right)}^{m}\right) \end{array}$$

Precision was calculated as follows: First, we store the acceptance/rejection evaluation of the *j*-th participant at the *i*th trial in the experiment in $z_{\left(j,i\right)}^{h}$, where *j* = 1, ⋯, 20. Second, we store the acceptance/rejection evaluation results of model *m* in the *i*-th trial of the pseudo-experiment for subject *j* in $z_{\left(j,i\right)}^{m}$, where *i* = 1, ⋯, 45 (*i* = 1, ⋯, 90 for both datasets). Third, we calculate the precision of model *m* in predicting the behavior of the *j*-th participant.

Precision Prec_*m*_ is calculated by counting the number of matches between the decisions of the participants and the model. One-sided tests were conducted for all model combinations.

## 4 Results and discussion

### 4.1 Hypothesis testing 1

**Figure 7:**
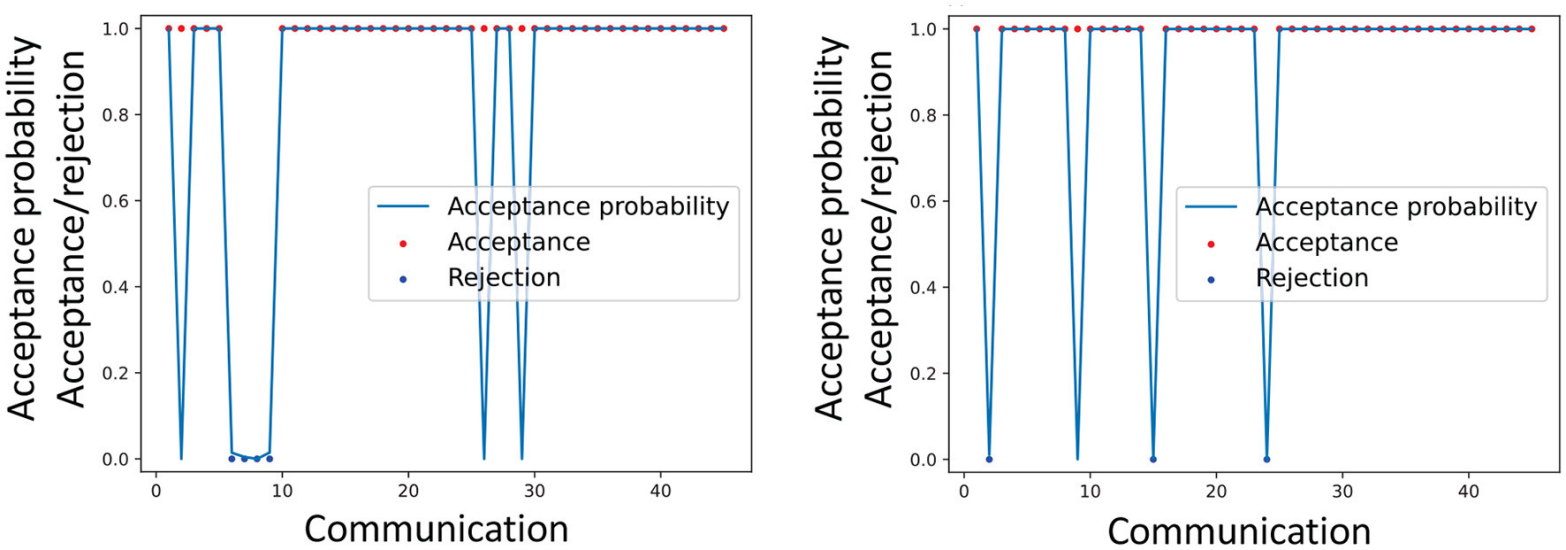
Example of the actual acceptance made by a participant and the inferred acceptance probabilities $r_{n}^{MH}$. Dataset 1 **(left)**, Dataset 2 **(right)**.

Figure 7 illustrates an example of the actual acceptance/rejection behavior of a participant and the inferred acceptance probabilities *r*^*MH*^. This suggests some coherence between $r_{n}^{MH}$ and the behavior of participants. This association was evaluated quantitatively and statistically.

**Figure 8:**
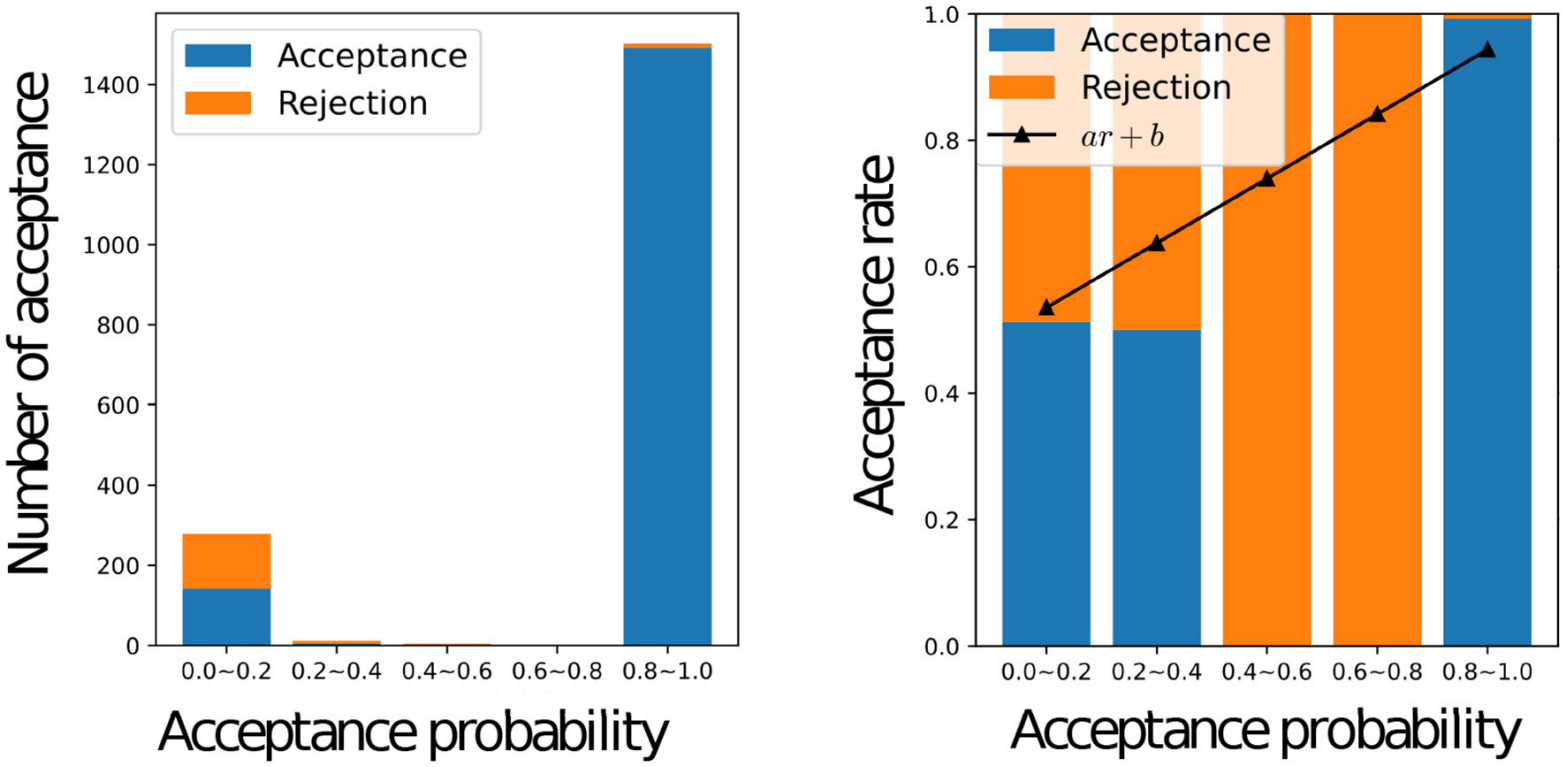
Relationship between the acceptance status of all participants and the inferred value of the acceptance probability Graphs of the number of accepted names for each inferred acceptance probability for all participants **(left)**, the actual acceptance rate for each inferred acceptance probability for all participants, and the graph of *y* = *ar* + *b* with weights “*a*” and bias “*b*” estimated by linear regression **(right)**.

**Figure 9:**
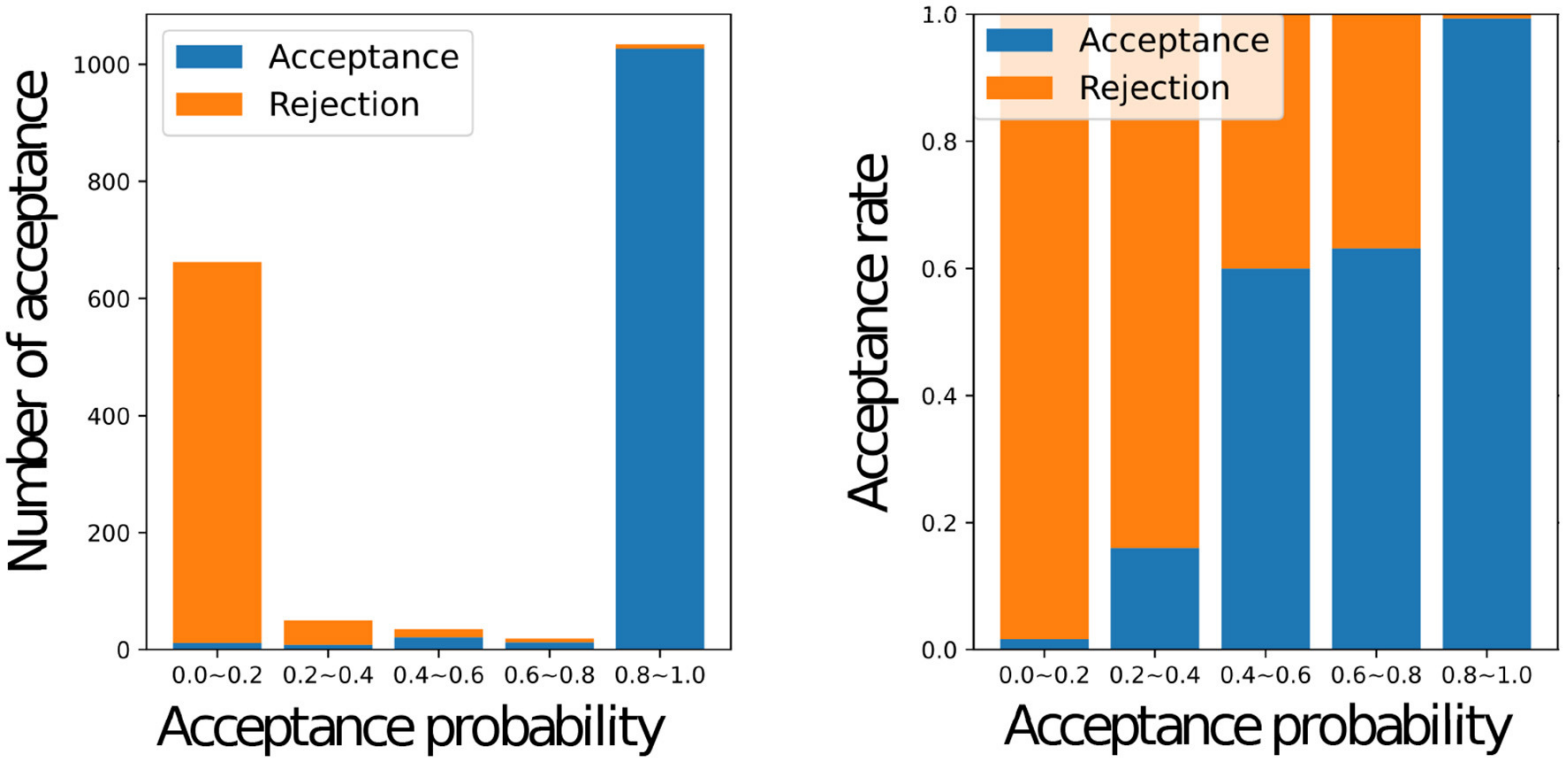
Relationship between the acceptance status of the computer and the value of the acceptance probability Graphs of the number of accepted names for each acceptance probability for the computer **(left)**, the actual acceptance rate for each acceptance probability in the simulation **(right)**.

Figure 8 shows a histogram of the number of accepted stimuli for each acceptance rate (left) and the actual acceptance rate for each acceptance rate with a graph of *y* = *ar*+*b* using the estimated weights *a* and bias *b* (right) for all the participants, where *a* = 0.5105 and *b* = 0.4842. When the inferred acceptance rate was high, the actual acceptance rate by humans was also high. However, the actual probability of acceptance was higher than *r*^*MH*^ when *r*^*MH*^ was low. It was rare for the inferred acceptance rate, $r_{n}^{MH}$ to assume intermediate values between 0.2 and 0.8. This bias is inherent in the nature of the MHNG and does not reflect the characteristics of human participants. In the MH algorithm, acceptance probability is determined by the ratio of one probability to another, which often results in values close to 0 or 1. For further clarification, Figure 9 shows the acceptance rate as observed in computer simulations for comparison.

Here, we discuss the results of the hypotheses tests. First, we examine the results of Test 1. The estimated parameters for Datasets 1 and 2 are shown in Table 4. The hypothesis tests for each and both datasets for all subjects were rejected at a significance level of 0.001. Therefore, the null hypothesis was rejected, indicating that the model using acceptance probability computed by the MH algorithm is a significantly better predictor of human behavior than the model using a constant probability of acceptance.

**Table 4:** Parameters *a,b* estimated and *p*-values on the data of each subject and the data aggregated over all participants for each dataset.

	**Dataset 1 (hard)**			**Dataset 2 (easy)**		
	*a*	*b*	*P*_*a*_(*a*)	*a*	*b*	*P*_*a*_(*a*)
1	0.6195	0.3749	< 0.001	0.6698	0.0000	< 0.001
2	0.2167	0.7832	0.011	0.2348	0.7938	0.002
3	0.4305	0.6085	0.022	0.3260	0.7223	0.066
4	0.3903	0.6557	0.009	0.7046	0.2499	< 0.001
5	0.2253	0.7771	0.011	−0.0002	1.0000	1.0
6	0.1227	0.8783	0.034	−0.0004	1.0002	1.0
7	0.5659	0.4285	0.001	0.7047	0.2496	< 0.001
8	0.4181	0.5552	0.001	0.0600	1.0007	0.432
9	0.4320	0.5779	0.002	0.3277	1.0001	1.0
10	−0.0001	1.0002	1.0	0.5000	0.5000	< 0.001
11	0.2608	0.7446	0.016	0.4816	0.4927	0.006
12	0.3551	0.6572	< 0.001	−0.0005	1.0071	1.0
13	0.7148	0.2846	< 0.001	0.4333	0.5766	0.002
14	0.2319	0.7682	0.012	−0.0005	1.0004	1.0
15	0.5850	0.3999	0.001	0.9187	0.0000	< 0.001
16	0.3537	0.6585	0.002	−0.0002	1.0001	1.0
17	0.8088	0.1383	< 0.001	0.5000	0.5000	< 0.001
18	−0.0003	1.0001	1.0	0.5000	0.5000	< 0.001
19	0.8642	0.1356	< 0.001	0.8971	0.0757	< 0.001
20	0.4477	0.2798	0.003	0.7933	0.0974	< 0.001
All	0.4910	0.5030	< 0.001	0.5478	0.4476	< 0.001
	**ALL dataset**					
	*a*		*b*		*P*_*a*_(*a*)	
All	0.5105		0.4842		< 0.001	

For more detail, we examined each participant's behavior. The *p*-values *P*_*a*_(*a*) for each subject, as presented in Table 4, indicate that the null hypothesis was rejected at the 0.05 significance level for most subjects. Specifically, exceptions were found for two participants, namely 10 and 18, in dataset 1. In dataset 2, all results were rejected at the 0.05 significance level except for the results for participants 5, 6, 9, 12, 14, and 16. Overall, the null hypothesis was rejected in most cases.

### 4.2 Hypothesis testing 2

Here, we examine the results of Test 2. Table 5 shows the *p*-values obtained from the *U*-tests conducted for each combination of models. The row for **MH** (i.e., *m* = 2) in Table 5 shows that the null hypothesis was rejected for all the models. The model using the MH algorithm was the closest to the behaviors of participants among the models compared in this study. We also individually performed tests on data from each participant. Table 6 presents the results. For each participant, **MH** outperformed the other models in predicting behavior in all cases, except for six participants in **Constant** and one in **Subtraction**. For the six participants, **MH** did not significantly outperform **Constant**, and for one participant, **MH** did not significantly outperform **Subtraction**. We tested the data for each participant separately and for each dataset. Tables 7, 8 list the results. Looking at the **MH** (i.e., *m* = 2) row in Table 7, **MH** outperformed the other models in all cases except seven for **Constant** and one for **Numerator**. Looking at the **MH** (i.e., *m* = 2) row in Table 8, **MH** outperformed the other models in all cases, except five for **Constant**. Based on these results, we argue that the acceptance probability derived from the MH algorithm is, to some extent, consistent with the acceptance/rejection judgment probabilities exhibited by humans.

**Table 5:** *p*-value for *U*-test for each model combination for all participants.

***m*\*m*^′^**	**Constant**	**MH**	**Numerator**	**Subtraction**	**Binary**
Constant	—	1.000	< 0.001	< 0.001	< 0.001
MH	< 0.001	—	< 0.001	< 0.001	< 0.001
Numerator	1.000	1.000	—	< 0.001	0.003
Subtraction	1.000	1.000	1.000	—	1.000
Binary	1.000	1.000	0.997	< 0.001	—

**Table 6:** Number of participants whose behavior resulted in the rejection of the null hypothesis for each pair of models.

***m*\*m*^′^**	**Constant**	**MH**	**Numerator**	**Subtraction**	**Binary**
Constant	—	4	12	18	12
MH	14	—	20	19	20
Numerator	6	0	—	20	7
Subtraction	2	0	0	—	0
Binary	6	0	1	20	—

**Table 7:** Number of participants whose behavior resulted in the rejection of the null hypothesis for each pair of models in Dataset 1.

***m*\*m*^′^**	**Constant**	**MH**	**Numerator**	**Subtraction**	**Binary**
Constant	—	6	12	18	12
MH	13	—	19	20	20
Numerator	6	0	—	20	8
Subtraction	2	0	0	—	0
Binary	5	0	4	20	—

**Table 8:** Number of participants whose behavior resulted in the rejection of the null hypothesis for each pair of models in Dataset 2.

***m*\*m*^′^**	**Constant**	**MH**	**Numerator**	**Subtraction**	**Binary**
Constant	—	3	10	18	9
MH	15	—	20	20	20
Numerator	6	0	—	20	9
Subtraction	2	0	0	—	0
Binary	4	0	4	20	—

The experimental results suggested that human behavior in the JA-NG follows the MH algorithm. Consequently, this result suggests that symbol emergence in the JA-NG among people may be attained by performing decentralized Bayesian inference, i.e., collective predictive coding (Taniguchi, 2023).

## 5 Conclusion

In this study, we conducted a communication experiment on symbol emergence, in which participants played the JA-NG in pairs. We compared the acceptance decisions of human participants with those of the computational models and confirmed that the acceptance probability of the model based on the MH algorithm predicted human behavior significantly better than the constant probability acceptance model. Additionally, the MH-based model outperformed the other four comparative computational models in terms of predicting the behavior of participants through two statistical tests. Consequently, the model using the MH algorithm was found to be suitable for explaining human acceptance behavior in the JA-NG.

This suggests that the MHNG, which was studied computationally as a constructive approach to human symbol emergence, is a reasonable model for explaining symbol emergence in computational agents and human groups. This finding also supports the collective predictive coding hypothesis, which argues that symbol emergence in human society can be regarded as a decentralized Bayesian inference of a prior variable shared among people (Taniguchi, 2023; Taniguchi et al., 2023). To advance our understanding of the human acceptance evaluation in the JA-NG and the dynamics of symbol emergence among people, future studies should gather more evidence by conducting experiments in diverse scenarios to test whether they follow the MH algorithm.

The limitations of the current experiment, as well as potential future extensions, are outlined below.

In the experiment presented in this paper, the number of participants in the JA-NG was limited to two. Theoretically, the MHNG model is capable of accommodating multiple agents. Inukai et al. (2023) proposed the Recursive MHNG, demonstrating its potential to handle *N*-agent scenarios. Additionally, in our experiment, the same object, i.e., a color image, was shown simultaneously to both participants. Yet, the original computational model facilitated symbol emergence among two agents observing a single object from distinct perspectives (Inukai et al., 2023; Taniguchi et al., 2023). Given the multi-agent setting and diverse perspectives in a real environment, incorporating these factors into an experimental semiotic study based on the JA-NG is a promising avenue for extension.

From the viewpoint of MHNG theory, there are still several limitations. Although emergent communication research currently places significant emphasis on the emergence of compositional linguistic structures with syntax (Lazaridou and Baroni, 2020), the MHNG only rigorously addresses the emergence of categorical symbols in a mathematical sense. An extension is needed to capture more complex linguistic structures. Also, as mentioned in Section 3.4, the current theory does not account for social and other cognitive aspects. Incorporating these considerations is another direction we plan to explore. In addition, the JA-NG assumes joint attention. While joint attention is not restricted to an external object, extending the theory to non-external objects, such as emotions, is another avenue for future exploration (Taniguchi, 2021).

Exploring symbol emergence in a human-agent mixed system is a future challenge worth pursuing. Because we obtained evidence supporting the prediction of the behavior of human participants using the MH algorithm, we could approximate human behavior as a computational agent following the MH algorithm. Based on this approximation, we can theoretically model and analyze a mixed system involving a human participant and a computer agent.

## Data availability statement

The datasets presented in this study can be found in online repositories. The names of the repository/repositories and accession number(s) can be found at: https://github.com/OkumuraRyotaDesu/Subject-Experimental-result-Data.

## Ethics statement

The studies involving humans were approved by the Research Ethics Committee of Ritsumeikan University. The studies were conducted in accordance with the local legislation and institutional requirements. The participants provided their written informed consent to participate in this study.

## Author contributions

RO designed the study, collected data, conducted the experiment, and wrote the manuscript. TT contributed to the key idea of this study, performed data analysis and interpretation, and contributed to the writing of the manuscript. AT and YH critically reviewed the manuscript and assisted in its preparation. All authors approved the final version of the manuscript and agreed to be accountable for all aspects of the work, ensuring that any questions related to the accuracy or integrity of any part of the work are appropriately investigated and resolved.

## Footnotes

^1^Pillow (PIL Fork) 8.4.0: https://pillow.readthedocs.io/en/stable/.

^2^This is because it is difficult to analytically determine the distribution that the estimated statistics *a* follow. We could not perform conventional tests such as the *t*-test.

## References

[B1] Bishop C. M. Nasrabadi N. M. (2006). Pattern Recognition and Machine Learning, Vol. 4. New York. NY: Springer.

[B2] Bouchacourt D. Baroni M. (2019). Miss tools and MR fruit: emergent communication in agents learning about object affordances. arXiv [preprint]. 10.48550/arXiv.1905.11871

[B3] Cangelosi A. Parisi D. (1998). The emergence of a'language'in an evolving population of neural networks. Conn. Sci. 10, 83–97. 10.1080/095400998116512

[B4] Choi E. Lazaridou A. de Freitas N. (2018). “Compositional obverter communication learning from raw visual input,” in The International Conference on Learning Representation (Vancouver, BC). Available online at: https://iclr.cc/archive/www/doku.php%3Fid=iclr2018:main.html

[B5] Cornish H. (2010). Investigating how cultural transmission leads to the appearance of design without a designer in human communication systems. Interact. Stud. 11, 112–137. 10.1075/is.11.1.02cor

[B6] Evtimova K. Drozdov A. Kiela D. Cho K. (2018). “Emergent communication in a multi-modal, multi-step referential game,” in Conference on Learning Representations (ICLR) (Vancouver, BC: Vancouver Convention Center). Available online at: https://iclr.cc/archive/www/doku.php%3Fid=iclr2018:main.html

[B7] Friston K. (2010). The free-energy principle: a unified brain theory? Nat. Rev. Neurosci. 11, 127–138. 10.1038/nrn2787 20068583

[B8] Friston K. Moran R. J. Nagai Y. Taniguchi T. Gomi H. Tenenbaum J.. (2021). World model learning and inference. Neural Netw. 144, 573–590. 10.1016/j.neunet.2021.09.011 34634605

[B9] Galantucci B. (2005). An experimental study of the emergence of human communication systems. Cogn. Sci. 29, 737–767. 10.1207/s15516709cog0000_34 21702792

[B10] Gluyas H. (2015). Effective communication and teamwork promotes patient safety. Nurs. Standard 29, 50. 10.7748/ns.29.49.50.e10042 26243123

[B11] Hagiwara Y. Furukawa K. Taniguchi A. Taniguchi T. (2022). Multiagent multimodal categorization for symbol emergence: emergent communication via interpersonal cross-modal inference. Adv. Robot. 36, 239–260. 10.1080/01691864.2022.2029721

[B12] Hagiwara Y. Kobayashi H. Taniguchi A. Taniguchi T. (2019). Symbol emergence as an interpersonal multimodal categorization. Front. Robot. AI 6, 134. 10.3389/frobt.2019.00134 33501149 PMC7805687

[B13] Hastings W. K. (1970). Monte Carlo sampling methods using Markov chains and their applications. Biometrika 57, 97–109. 10.1093/biomet/57.1.97

[B14] Havrylov S. Titov I. (2017). Emergence of language with multi-agent games: learning to communicate with sequences of symbols. Adv. Neural Inf. Process. Syst. 30. Available online at: https://openreview.net/forum?id=SkaxnKEYg

[B15] Healey P. G. Swoboda N. Umata I. King J. (2007). Graphical language games: Interactional constraints on representational form. Cogn. Sci. 31, 285–309. 10.1080/15326900701221363 21635298

[B16] Hohwy J. (2013). The Predictive Mind. Oxford: Oxford University Press. 10.1093/acprof:oso/9780199682737.001.0001

[B17] Inukai J. Taniguchi T. Taniguchi A. Hagiwara Y. (2023). Recursive metropolis-hastings naming game: symbol emergence in a multi-agent system based on probabilistic generative models. Front. Artif. Intell. 6, 1229127. 10.3389/frai.2023.1229127 37920571 PMC10619661

[B18] Kirby S. (2002). Natural language from artificial life. Artif. Life 8, 185–215. 10.1162/106454602320184248 12171637

[B19] Kirby S. Cornish H. Smith K. (2008). Cumulative cultural evolution in the laboratory: an experimental approach to the origins of structure in human language. Proc. Natl. Acad. Sci. 105, 10681–10686. 10.1073/pnas.0707835105 18667697 PMC2504810

[B20] Kottur S. Moura J. M. Lee S. Batra D. (2017). Natural language does not emerge ‘naturally' in multi-agent dialog. arXiv [preprint]. 10.48550/arXiv.1706.08502

[B21] Lazaridou A. Baroni M. (2020). Emergent multi-agent communication in the deep learning era. arXiv [preprint]. 10.48550/arXiv.2006.02419

[B22] Lazaridou A. Peysakhovich A. Baroni M. (2017). “Multi-agent cooperation and the emergence of (natural) language,” in International Conference on Learning Representations (ICLR). Toulon. Available online at: https://iclr.cc/archive/www/2017.html

[B23] Lewis D. (2008). Convention: A Philosophical Study. Hoboken, NJ: John Wiley and Sons.

[B24] Mu J. Goodman N. (2021). Emergent communication of generalizations. Adv. Neural Inf. Process. Syst. 34, 17994–18007. Available online at: https://proceedings.neurips.cc/paper_files/paper/2021/file/9597353e41e6957b5e7aa79214fcb256-Paper.pdf

[B25] Navarro D. J. Perfors A. Kary A. Brown S. D. Donkin C. (2018). When extremists win: cultural transmission via iterated learning when populations are heterogeneous. Cogn. Sci. 42, 2108–2149. 10.1111/cogs.12667 30062733

[B26] Quinn M. (2001). “Evolving communication without dedicated communication channels,” in Advances in Artificial Life: 6th European Conference, September 10-14, 2001. Proceedings (Cham: Springer), 357–366. 10.1007/3-540-44811-X_38

[B27] Ren Y. Guo S. Labeau M. Cohen S. B. Kirby S. (2020). “Compositional languages emerge in a neural iterated learning model,” in International Conference on Learning Representations (ICLR). virtual. Available online at: https://iclr.cc/virtual_2020/index.html

[B28] Roberts G. (2010). An experimental study of social selection and frequency of interaction in linguistic diversity. Interact. Stud. 11, 138–159. 10.1075/is.11.1.06rob

[B29] Scott-Phillips T. C. Kirby S. Ritchie G. R. (2009). Signalling signalhood and the emergence of communication. Cognition 113, 226–233. 10.1016/j.cognition.2009.08.009 19740461

[B30] Steels L. (1999). The spontaneous self-organization of an. Mach. Intell. 15, 205. 10.1093/oso/9780198538677.003.0011

[B31] Steels L. (2015). The Talking Heads experiment: Origins of Words and Meanings. Berlin: Language Science Press. 10.26530/OAPEN_559870

[B32] Steels L. Belpaeme T. (2005). Coordinating perceptually grounded categories through language: a case study for colour. Behav. Brain Sci. 28, 469–488. 10.1017/S0140525X05000087 16209771

[B33] Taniguchi T. (2021). “On parallelism in music and language: a perspective from symbol emergence systems based on probabilistic generative models,” in International Symposium on Computer Music Multidisciplinary Research (New York, NY: Springer), 9–25. 10.1007/978-3-031-35382-6_2

[B34] Taniguchi T. (2023). Collective predictive coding hypothesis: Symbol emergence as decentralized bayesian inference. PsyArXiv [preprint]. 10.31234/osf.io/d2ty6 PMC11300318 39109321

[B35] Taniguchi T. Nagai T. Nakamura T. Iwahashi N. Ogata T. Asoh H.. (2016). Symbol emergence in robotics: a survey. Adv. Robot. 30, 706–728. 10.1080/01691864.2016.1164622

[B36] Taniguchi T. Nakamura T. Suzuki M. Kuniyasu R. Hayashi K. Taniguchi A.. (2020). Neuro-serket: development of integrative cognitive system through the composition of deep probabilistic generative models. New Gener. Comput. 38, 23–48. 10.1007/s00354-019-00084-w

[B37] Taniguchi T. Ugur E. Hoffmann M. Jamone L. Nagai T. Rosman B.. (2018). Symbol emergence in cognitive developmental systems: a survey. IEEE Trans. Cogn. Dev. Syst. 11, 494–516. 10.1109/TCDS.2018.2867772

[B38] Taniguchi T. Yoshida Y. Matsui Y. Le Hoang N. Taniguchi A. Hagiwara Y.. (2023). Emergent communication through metropolis-hastings naming game with deep generative models. Adv. Robot. 37, 1–17. 10.1080/01691864.2023.2260856 PMC10864618 38356917

